# Protective effect of 1, 8-cineole (eucalyptol) against lead-induced liver injury by ameliorating oxidative stress and inflammation and modulating TLR4/MyD88/NF-κB signaling

**DOI:** 10.22038/ijbms.2024.78448.16964

**Published:** 2024

**Authors:** Mojdeh Abdollahi, Masoumeh Asle-Rousta, Sanaz Mahmazi

**Affiliations:** 1 Department of Genetics, Zanjan Branch, Islamic Azad University, Zanjan, Iran; 2 Department of Physiology, Zanjan Branch, Islamic Azad University, Zanjan, Iran

**Keywords:** Cytokines, Eucalyptol, Lead acetate, Liver, Oxidative stress, Toll-like receptor 4

## Abstract

**Objective(s):**

This study was conducted to explore the impact of 1, 8-cineole (eucalyptol) on the biochemical, molecular, and histological changes caused by lead acetate in the liver of adult male Wistar rats. The research also investigated the potential involvement of the TLR4 signaling pathway in this effect.

**Materials and Methods:**

Rats were orally administered lead acetate (25 mg/kg-day) for 14 consecutive days and received 1, 8-cineole (100 mg/kg-day) during the same period.

**Results:**

1, 8-cineole prevented an increase in the malondialdehyde level, a decrease in the glutathione level, and a decrease in the activity of superoxide dismutase and glutathione peroxidase enzymes in the liver of rats treated with lead acetate. This monoterpene also prevented an increase in the expression of pro-inflammatory cytokines and significantly reduced the infiltration of inflammatory cells in the liver parenchyma. Additionally, 1, 8-cineole discouraged the increase in toll-like receptor 4 (TLR4), myeloid differentiation primary response 88 (MyD88), and nuclear factor kappa B (NF-κB) expression in the liver and stopped a rise in serum AST and ALT enzymes.

**Conclusion:**

1, 8-cineole can prevent liver damage caused by lead acetate by reducing oxidative stress and inflammation. This hepatoprotection is probably achieved by inhibiting TLR4/MyD88/NF-κB signaling.

## Introduction

Lead, a common environmental pollutant, can enter the human body through various means, such as water, food, and air. Due to its widespread use in industry and indestructibility, lead pollution is a serious issue in many countries, raising serious concerns for human health (1, 2). Once inside the body, lead accumulates in organs such as the liver and kidneys (3). Autopsy studies of individuals exposed to lead have revealed that approximately 33% of the absorbed lead is stored in the liver (4). Research has shown that lead can increase the production of reactive oxygen species (ROS) while weakening the anti-oxidant system in various organs, including the liver, kidneys, and brain (5, 6). Lead also plays an essential role in inflammation, resulting in the production of primary mediators of inflammatory processes such as tumor necrosis factor (TNF)-α, interleukin (IL)-1, IL-2, IL-6, IL-8, IL-15, and interferon-gamma (7, 8). Oxidative stress and inflammation caused by lead can damage liver cells and disrupt this organ’s function. Currently, natural compounds are being studied for their anti-oxidant potential to reduce lead toxicity in the liver (9). 

1, 8-cineole (1,3,3-trimethyl-2-oxabicyclo[2.2.2]octane), also known as eucalyptol, is a type of monoterpene present in various plants, including eucalyptus, rosemary, and camphor (10). This compound is safe and does not cause toxicity in rats unless taken in doses higher than 600 mg/kg (11), while its therapeutic effects can be observed at much lower doses (10). 1,8-Cineole has been proven to be beneficial in treating respiratory problems, including rhinosinusitis, bronchitis, asthma, and chronic obstructive pulmonary disorder (12). Additionally, it has cardioprotective (13), neuroprotective (14), and antidiabetic effects (15). Its protective effects are mainly due to its ability to reduce oxidative stress and inflammation and regulate nuclear factor kappa B (NF-κB) and nuclear factor erythroid 2-related factor 2 (16).

Based on this information, a hypothesis was proposed regarding the hepatoprotective effects of 1, 8-cineole against lead-induced liver damage. To test this hypothesis, we investigated the effects of 1, 8-cineole on oxidative stress, inflammation, and histopathological changes in the liver of rats receiving lead acetate. Additionally, the study examined the possible effect of 1, 8-cineole on the toll-like receptor 4 (TLR4)/myeloid differentiation primary response 88 (MyD88)/NF-κB signaling pathway, which plays a crucial role in the initiation and progression of liver-related diseases (17). 

## Materials and Methods

***Experimental design***

Twenty-four male Wistar rats weighing between 200–220 g were purchased from the Neuroscience Research Center of Shahid Beheshti University of Medical Sciences, Iran. The rats were kept under standard conditions, which included a temperature range of 23–25 °C, 50–55% relative humidity, 12/12 hr light and dark cycle, and standard nutrition. They also had *ad libitum* access to water. The animal protocol was conducted following the approval of the Ethical Committee for Animal Experiments of Islamic Azad University, Zanjan branch (Approval number: IR.IAU.Z.REC.1402.048). After the rats were acclimatized to the laboratory conditions, they were randomly divided into four groups, each consisting of 6 rats. 

The first was the Control group, which did not receive any treatment. The second group, called the Cineole group, was given 1, 8-cineole orally at a dose of 100 mg/kg-day (18) for 14 consecutive days. The third group, the Lead group, was treated with lead acetate orally at a dose of 25 mg/kg-day for 14 days (19). The fourth group, the Lead-Cineole group, was given both lead acetate and 1, 8-cineole with the mentioned doses for 14 days. Lead acetate and 1, 8-cineole were purchased from Sigma (USA).

After this period of 14 days, following the ketamine (50 mg/kg)-xylazine (10 mg/kg) anesthesia (20), blood was collected from the left ventricle of the animals, and their livers were removed for further studies. The liver was analyzed biochemically to determine the levels of malondialdehyde (MDA), glutathione (GSH), and the activity of superoxide dismutase (SOD) and glutathione peroxidase (GPx). Additionally, molecular studies were conducted to measure the mRNA level of TNF-α, IL-1β, IL-6, TLR4, MyD88, and NF-κB by real-time polymerase chain reaction (PCR), and histological studies were carried out.

***Biochemical studies***

*Estimation of liver enzyme activity*

The levels of alanine transaminase (ALT) and aspartate transaminase (AST) in the serum were measured using Bionik enzyme kits (Iran). The tests were performed according to the instructions provided.

*Homogenization of liver samples*

The liver samples were cut into small pieces and blended in Tris-HCl buffer (25 mM, pH 7.5) using a homogenizer to create a liver homogenate. The resulting 10% (w/v) liver homogenate was centrifuged at 12,000 rpm for 15 min at 4 °C. To measure the protein concentration of each extract, the Lowry method (21) was used with bovine serum albumin as the standard.

*Assessing the levels of MDA*

The concentration of MDA in a sample was measured using the Arsam Fara Zist lipid peroxidation assay kit, following the provided instructions. A pink solution made by combining MDA and thiobarbituric acid (TBA) was used to determine the amount of MDA. The test involved mixing 250 μl of supernatant with 500 μl of 10% trichloroacetic acid solution and incubating the mixture at 95 °C for 5 min. After centrifugation at 14000 g for 5 min, the 500 μl supernatant of each tube was mixed with 250 μl of 0.67% TBA solution in new tubes. The mixtures were kept at 95 °C for 30 min and then cooled to room temperature. The absorbance of each tube was measured against the blank solution at 532 nm. Finally, the concentration of MDA was expressed as nmol/mg.

*Measuring GSH*

To measure the amount of GSH, a mixture of 100 μl of supernatant, 300 μl of phosphate-buffered saline, and 100 μl of 0.8% sulfosalicylic acid was prepared. Then, the mixture was cooled on ice for 10 min and centrifuged at 12000 g for 5 min. After that, 400 μl of the supernatant was mixed with 400 μl of 300 mM tris buffer and 100 μl of 5, 5′-dithiobisnitro benzoic acid (0.04 mg/ml) (22). Finally, the absorbance was measured at 412 nm against the blank solution, and the GSH content was expressed as nmol/mg.

*Determination of SOD and GPx activities*

The activity of the SOD enzyme in homogeneous tissue samples was determined using the Nasdox™ assay kit from Navand Salamat, Iran. This kit prevents pyrogallol from oxidizing and measures the time it takes to oxidize at a certain amount. By comparing the inhibition of pyrogallol oxidation at a specific time with a control concentration of SOD, the activity of SOD in an unidentified sample can be determined. First, 50 μl of the supernatant was mixed with 200 μl of reagent 1 and 50 μl of reagent 2. Then, it was incubated for 5 min at room temperature and kept away from light. After the incubation period, the absorbance was measured using a spectrophotometer at 405 nm against the blank. 

Similarly, the Nagpix™ kit from Navand Salamat (Iran) was used to measure GPx activity. This kit works by measuring the consumption of nicotinamide adenine dinucleotide phosphate (NADPH) by the GPx enzyme, thereby determining the enzyme’s activity level. For this test, 50 μl of supernatant was mixed with 40 μl of reagent 1. After incubating for 15 min at room temperature, 10 μl of reagent 2 was added and thoroughly mixed. Then, the optical absorbance was measured by a spectrophotometer at 340 nm against a blank.

The activity of SOD and GPx enzymes was expressed as U/mg.

*Investigating gene expression using* *real time-PCR*

The research also involved conducting gene expression analysis on liver tissue samples taken from five animals in each group. The Parstous RNA extraction kit was used to extract total mRNA, which was then checked for quality using the A260/A280 ratio and 260 nm wavelength. To create cDNA, the Easy cDNA Synthesis Kit from Parstous was utilized. The gene expression of each group was evaluated using the real-time PCR technique. Each vial was prepared with a final volume of 20 μl, containing 1 μl of reverse and forward primers, 2 μl of cDNA, 6 μl of double-distilled water, and 10 μl of qPCRBIO SyGreen Mix Lo-ROX by PCR BIOSYSTEMS (UK). The thermal cycling conditions involved a primary denaturation cycle (95 °C for 2 min), followed by 40 cycles of denaturation (95 °C for 30 sec), annealing (52 °C for 30 sec), and extension (72 °C for 20 sec). The relative gene expression was assessed using the 2^-ΔΔCT^ method (23) with glyceraldehyde-3-phosphate dehydrogenase (GAPDH) used as an internal control gene. The primer sequences are presented in Table 1.

*Histological examination*

The liver samples obtained from each animal were carefully examined under a microscope to identify any signs of pathology. First, the samples were fixed in 10% formalin and then embedded in paraffin. After that, the slices with a thickness of 5 µm were prepared and stained with hematoxylin-eosin (H&E). The primary objective was to determine the extent of cellular infiltration in the liver parenchyma. For this goal, ten fields from the liver of each animal were analyzed. Lobular inflammation was graded based on the extent of cellular infiltration. The following criteria were used: (-) for no infiltration, (+/-) for less than 5% of fields, (+) for less than 20% of fields, (++) for 20-60% of fields, and (+++) for more than 60% of fields (24).

***Statistical analysis***

The statistical analysis was performed using SPSS software. The data is presented as the mean ± standard error of the mean (SEM). One-way analysis of variance was used for statistical analysis with Tukey’s test for *post hoc* comparisons. If the *P*-value was less than 0.05, the result was significant.

## Results

***The effect of 1, 8-cineole on the change of AST and ALT levels in rats receiving lead acetate***

**Figure 1:**
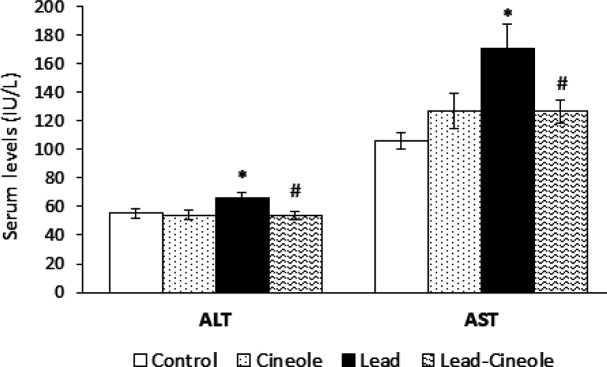
Effect of 1, 8-cineole on plasma levels of alanine aminotransferase (ALT) and aspartate aminotransferase (AST) in the serum of rats receiving lead acetate

In the Lead group, the levels of ALT and AST enzymes in the serum were significantly higher compared to the Control group (*P*=0.027 and *P*=0.021, respectively). Moreover, 1, 8-cineole caused a significant decrease in enzyme levels in the serum of rats belonging to the Lead-Cineole group compared to the Lead group (*P*=0.017 and *P*=0.018, respectively) (Figure 1). 

***Effect of 1, 8-cineole on oxidative stress induced by lead acetate in the liver***

**Figure 2:**
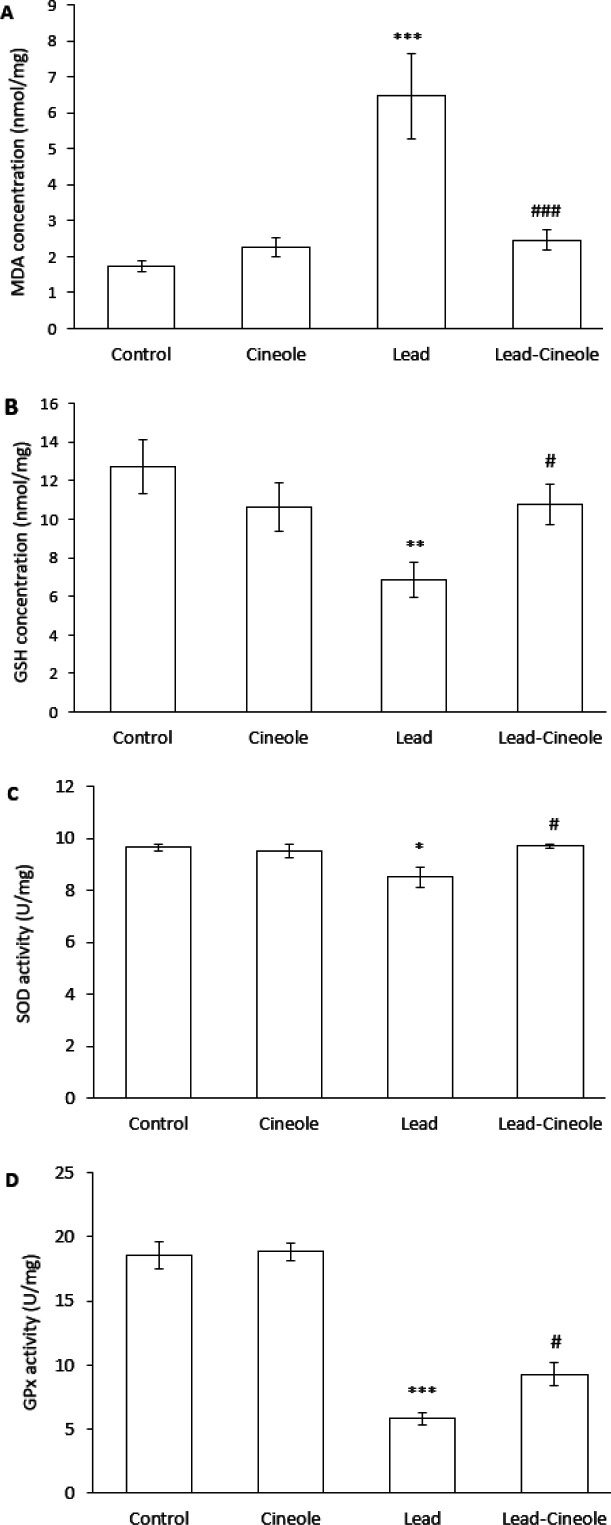
Effect of 1, 8-cineole on the level of (A) MDA and (B) GSH and the activity of (C) SOD and (D) GPx enzymes in the liver of rats receiving lead acetate

The administration of lead acetate to rats resulted in higher levels of MDA in their livers compared to the Control group (*P*=0.000). However, when the rats were treated with both 1, 8-cineole and lead acetate, the levels of MDA were significantly lower compared to the Lead group (*P*=0.000) (Figure 2A). 

Moreover, the content of GSH decreased significantly in the liver of the Lead group as compared to the Control group (*P*=0.007). However, in the Lead-Cineole group, GSH levels were significantly higher than in the Lead group (*P*=0.044) (Figure 2B).

The activities of SOD and GPx enzymes were significantly decreased in the liver of rats treated with lead acetate compared to the Control group (*P*=0.015 and *P*=0.000, respectively). Treatment with 1, 8-cineole significantly increased the activity of SOD (*P*=0.024) and GPx (*P*=0.018) in the liver of the Lead-Cineole group compared to the Lead group (Figure 2C, D).

The control group did not show significant differences in any of the factors related to this part of the study compared to the Cineole group (Figure 2).

***Effect of 1, 8-cineole on inflammation induced by lead acetate in the liver***

**Figure 3:**
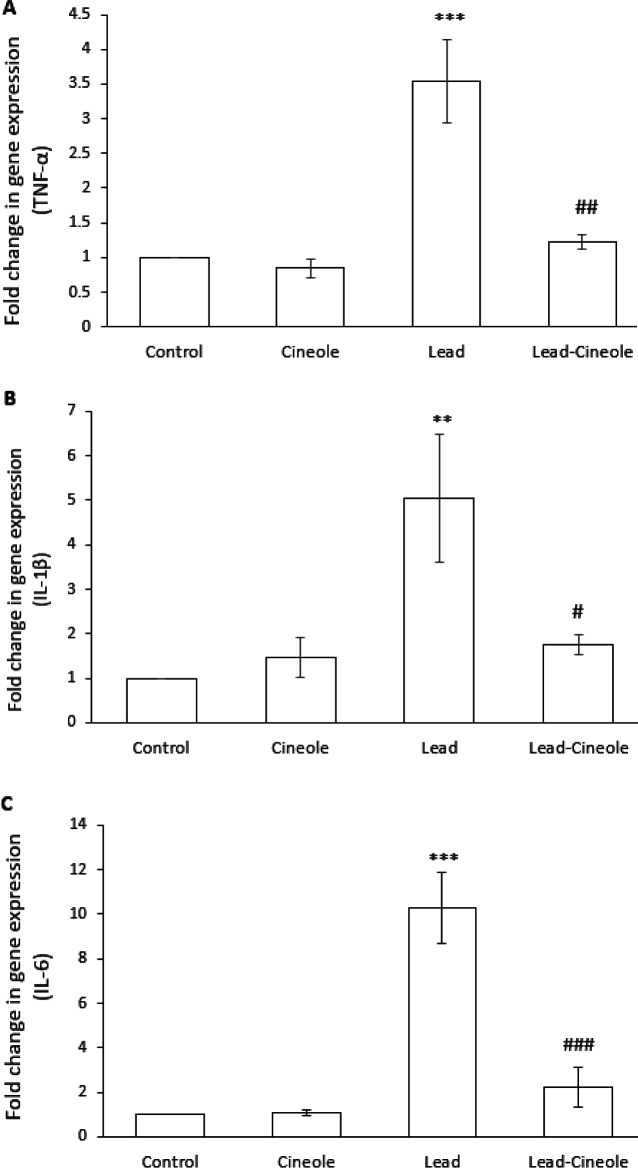
Effect of 1, 8-cineole on (A) TNF-α, (B) IL-1β, and (C) IL-6 mRNA expression in the livers of rats receiving lead acetate

The administration of lead acetate to rats increased the expression of pro-inflammatory factors in the liver. Specifically, the mRNA levels of TNF-α (*P*=0.000), IL-1β (*P*=0.006), and IL-6 (*P*=0.000) were significantly higher compared to the Control group. Conversely, the rats in Lead-Cineole group showed significantly lower expression of these inflammatory mediators compared to the Lead group (*P*=0.001, *P*=0.015, and *P*=0.000, respectively) (Figure 3). The expression of these factors in the Cineol group was not significantly different compared to the Control group.

***Effect of 1, 8-cineole on TLR4/MyD88/NF-κB signaling in the liver of rats receiving lead acetate***

**Figure 4:**
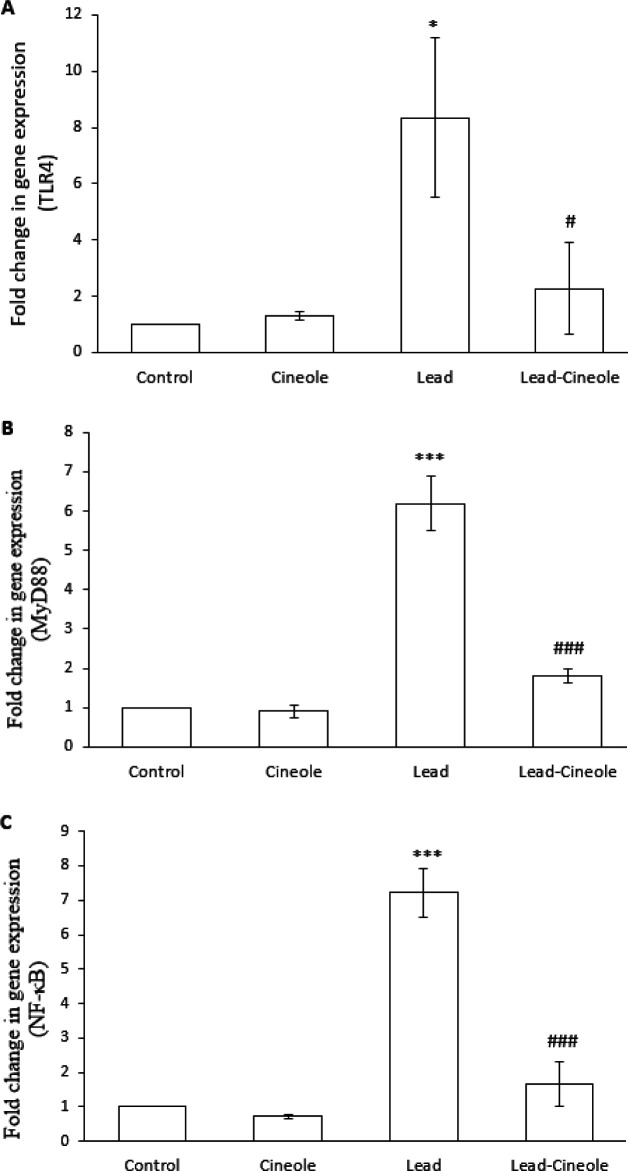
Effect of 1, 8-cineole on (A) TLR4, (B) MyD88, and (C) NF-κB mRNA expression in the liver of rats receiving lead acetate

Furthermore, lead acetate administration also resulted in a significant increase in the mRNA levels of TLR4, MyD88, and NF-κB in the liver of rats compared to the Control group (*P*=0.013, *P*=0.000, and *P*=0.000, respectively). However, when lead acetate and 1, 8-cineole were administered, these changes were prevented to a great extent. The expression of TLR4 (*P*=0.031), MyD88 (*P*=0.000), and NF-κB (*P*=0.000) in the Lead-Cineole group were significantly less compared to the Lead group (Figure 4). There was no significant difference in the expression of any of these factors in the Cineole group compared to the Control group.

***Effect of 1, 8-cineole on histopathological changes induced by lead acetate in the liver***

**Figure 5:**
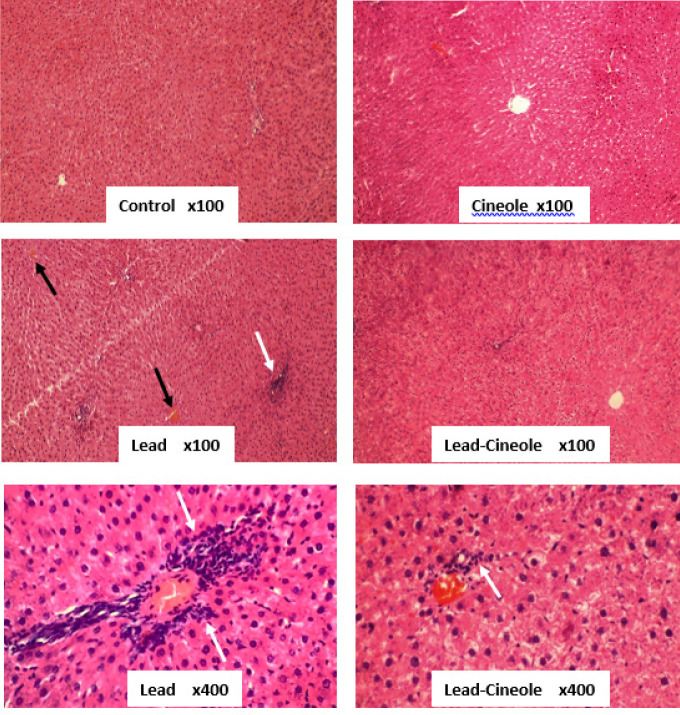
Effect of 1, 8-cineole on histopathological changes in the liver of rats receiving lead acetate

Upon microscopic examination, 5-μm sections stained with H&E revealed a significant infiltration of mononuclear inflammatory cells in the liver parenchyma of animals that received lead acetate (++). However, the rats in the Lead-Cineole group, which were treated with 1, 8-cineole for 14 days, showed significant prevention of this phenomenon (+). Moreover, there was no evidence of inflammatory cell infiltration in the liver tissue of the Control and Cineole groups (-). In addition, lead acetate caused disarrangement of hepatocytes and central venous congestion, while 1, 8-cineole treatment largely prevented these events in the liver of rats receiving lead (Figure 5). 

**Table 1:** Primer sequence of TNF-α, IL-6, IL-1β, TLR4, MyD88, NF-κB and GAPDH for rat

Gene	Primer (5' ─ 3')
TNF-α	F: CACGGGAGCCGTGACTGTA R: TCCAAGCGAACTTTATTTCTCTCA
IL-6	F: ACTATGAGGTCTACTCGGCAAACC R: ACAGTGAGGAATGTCCACAAACTG
IL-1β	F: TCAGGAAGGCAGTGTCACTCA R: TCCACGGGCAAGACATAGGT
TLR4	F: AGCCTTGAATCCAGATGAAAC R: ACAGCAGAAACCCAGATGAA
MyD88	F: GCATATGCCTGAGCGTTTCG R: TTCTGATGGGCACCTGGAGA
NF-κB	F: CATGGCAGACGACGATCCTT R: TGGAGTGAGTCAAAGCAGTATTCAA
GAPDH	F: GCTACACTGAGGACCAGGTTGTCT R: CCCAGCATCAAAGGTGGAA

Results are shown as means ± SEM; n = 6. * *P*<0.05 compared with the Control group and # *P*<0.05 compared with the Lead group.

Data are presented as mean ± SEM; n = 6. * *P*<0.05, ** *P*<0.01, *** *P*<0.001 compared with the Control group and # *P*<0.05, ### *P*<0.001 compared with the Lead group.

MDA: malondialdehyde; GSH: glutathione; SOD: superoxide dismutase; GPX: glutathione peroxidase

Data are presented as mean ± SEM; n = 5. ** *P*<0.01, *** *P*<0.001 compared with the Control group and # *P*<0.05, ## *P*<0.01, ### *P*<0.001 compared with the Lead group.

TNF: tumor necrosis factor; IL: interleukin

Data are presented as mean ± SEM; n = 5. * *P*<0.05, *** *P*<0.001 compared with the Control group and # *P*<0.05, ### *P*<0.001 compared with the Lead group

TLR4: toll-like receptor 4; MyD88: myeloid differentiation primary response 88; NF-κB: nuclear factor kappa B

Liver sections stained with hematoxylin & eosin (H&E) were examined microscopically. Inflammatory cells are indicated by white arrows and congestion by black arrows. Ten fields were examined from each rat's liver.

## Discussion

During the current research, lead acetate led to oxidative stress and an increase in the expression of pro-inflammatory factors in the liver of rats. It also caused histopathological changes in the liver, leading to a change in its function. As a result, the levels of AST and ALT enzymes in the bloodstream increased. These changes were associated with an increase in the expression of TLR4, MyD88, and NF-κB. The findings suggest that administering lead acetate at a dose of 25 mg/kg-day for 14 days can cause liver damage in rats, which is consistent with previous studies (8, 25).

To explore the possibility of preventing or reducing the harmful effects of lead, 1, 8-cineole was administered simultaneously during lead acetate consumption. The dosage of 1, 8-cineole was selected based on its ability to reduce oxidative stress in the brains of ischemia model rats (18) and the livers of rats exposed to a persistent environmental pollutant known as 2,3,7,8-tetrachlorodibenzo-p-dioxin (26). 

Biochemical examinations revealed that 1, 8-cineole prevents oxidative damage in the liver of rats who received lead acetate by enhancing the anti-oxidant system. Previous *in vitro* and *in vivo* studies have also reported that 1, 8-cineole can reduce ROS production and increase the endogenous anti-oxidant status (17, 27, 28). Environmental pollutants cause oxidative stress and damage organic molecules in hepatocytes, leading to structural and functional abnormalities in these cells. This interference with the pathogenesis of various liver diseases has led to an increasing interest in anti-oxidative treatments for preventing and treating liver diseases (29, 30). 

Oxidative stress plays a crucial role in the development and continuation of inflammation. Oxidants affect all stages of the inflammatory response by activating TLR4 in the cell membrane, which triggers processes that lead to cell inflammation. However, the formation of ROS is also considered  the cell’s response to TLR4 activation (31). Studies have shown that an increase in TLR4 mRNA expression strengthens oxidative stress and inflammation and ultimately results in liver damage. In TLR4^-/-^ mice, acetaminophen treatment causes a smaller reduction in the amount of GSH compared to wild-type mice and a smaller increase in the expression of inflammatory factors (32). Therefore, since 1, 8-cineole reduced oxidative stress in the liver, it was expected to prevent the increase in TLR4 expression, and the results of molecular tests confirmed our hypothesis. We recommend investigating the activity of TLR4 in the liver of rats from the Lead and Lead-Cineole groups. 

TLR4 is associated with MyD88 on the inner side of the membrane. MyD88 is a vital component for the activation of innate immunity by all the TLRs (33). MyD88 leads to the activation of two pathways: NF-κB and c-Jun N-terminal kinase (34). NF-κB is a transcription factor that is activated by TLR4 and cytokines like TNF-α and IL-1β. It plays an essential role in inducing and functioning pro-inflammatory genes (35). Research suggests that there is a vicious circle between the increase in the expression of pro-inflammatory factors and NF-κB, where each side reinforces the other. In our study, lead acetate increased the expression of pro-inflammatory factors and NF-κB, but rats treated with 1, 8-cineole for 14 days prevented these events. Additionally, 1, 8-cineole also prevented the increased expression of MyD88 in the liver of these rats. Therefore, the anti-inflammatory effect of 1, 8-cineole may be due to the inhibition of TLR4/MyD88/NF-κB signaling. The results of the study are consistent with Linghu *et al*.’s reports (36, 37), which showed that 1, 8-cineole can prevent the increase in the expression of IL-6, IL-8, and NF-κB caused by lipopolysaccharide in both *in vitro* and *in vivo *conditions. This monoterpene’s reducing effect on the expression of cytokines and TLR4 in human bronchial epithelial cells has also been proven (38).

Several studies have suggested that NF-κB has a pro-oxidant effect by activating pro-oxidant genes (39). Therefore, the decrease in oxidative stress observed in rats treated with Lead-Cineole could be related to the ability of 1,8-cineole to reduce the expression of NF-κB. It is recommended to explore the protein expression of NF-κB and its translocation into the nucleus.

The liver can experience an increase in inflammatory cytokines, which leads to an increase in the expression of cell adhesion molecules on the portal and/or sinusoidal endothelial cells. Additionally, the down-regulation of platelet endothelial cell adhesion molecules can cause infiltration of inflammatory cells toward hepatocytes (40). By histological evaluation of the liver tissue, we found that treatment with 1, 8-cineole reduced the infiltration of mononuclear inflammatory cells in the liver of rats receiving lead acetate. This reduction was predictable, as 1, 8-cineole can reduce the expression of inflammatory cytokines. Another study showed that 1, 8-cineole can also prevent the infiltration of inflammatory cells around the alveolar and small bronchi of a mouse model infected with the influenza virus (41).

ALT and AST are indicators for measuring liver damage. Following the disruption of cell membrane integrity, the levels of these enzymes in the blood increases. Furthermore, it is attributed to necrosis and elevated expression of enzymes (42). Here, 1, 8-cineole prevented an increase in ALT and AST enzymes in the Lead group. This finding is supported by Santos *et al*. (43), who showed that pretreatment of 1, 8-cineole, and dexamethasone in rats discouraged the increase of AST level and TNF-α concentration in the serum. It is important to note that the effectiveness of 1, 8-cineole in preventing an increase in ALT and AST enzymes is dose-dependent. A three-day treatment with 1, 8-cineole at a dose of 800 mg/kg increased the ALT levels in mice that received thioacetamide (44). 

There is limited research conducted on the impact of 1, 8-cineole on the liver. Ciftci *et al*. (25) demonstrated that administering this monoterpene to rats exposed to 2,3,7,8-tetrachlorodibenzo-p-dioxin can increase the activity of catalase, SOD, and GPx, as well as the level of GSH, which helps to reduce oxidative stress in the liver. The study also revealed that 1, 8-cineole’s anti-oxidant effect is more significant when administered over 60 days compared to 30 days. Recently, the protective effect of this compound on cisplatin-induced oxidative stress and histopathological damage in the liver has also been reported (45).  Our research strengthened the hypothesis of hepatoprotection of 1, 8-cineole by conducting molecular, biochemical, and histological studies.

## Conclusion

Monoterpene 1, 8-cineole can prevent liver damage caused by lead acetate in rats. This is achieved by reducing oxidative stress and inflammation, inhibiting TLR4/MyD88/NF-κB signaling, and preventing histological changes.

## References

[1] Assi MA, Hezmee MN, Sabri MY, Rajion MA (2016). The detrimental effects of lead on human and animal health. Vet World.

[2] Karrari P, Mehrpour O, Abdollahi M (2012). A systematic review on status of lead pollution and toxicity in Iran; Guidance for preventive measures. DARU J Pharm Sci.

[3] Takano T, Okutomi Y, Mochizuki M, Ochiai Y, Yamada F, Mori M (2015). Biological index of environmental lead pollution: Accumulation of lead in liver and kidney in mice. Environ Monit Assess.

[4] Mudipalli A (2007). Lead hepatotoxicity & potential health effects. Indian J Med Res.

[5] Matović V, Buha A, Ðukić-Ćosić D, Bulat Z (2015). Insight into the oxidative stress induced by lead and/or cadmium in blood, liver and kidneys. Food Chem Toxicol.

[6] Neamatallah WA, Sadek KM, El-Sayed YS, Saleh EA, Khafaga AF (2022). 2, 3-Dimethylsuccinic acid and fulvic acid attenuate lead-induced oxidative misbalance in brain tissues of Nile tilapia Oreochromis niloticus. Environ Sci Pollut Res Int.

[7] Metryka E, Chibowska K, Gutowska I, Falkowska A, Kupnicka P, Barczak K (2018). Lead (Pb) exposure enhances expression of factors associated with inflammation. Int J Mol Sci.

[8] Abdel-Emam RA, Ali MF (2022). Effect of L-carnitine supplementation on lead acetate-induced liver cell apoptosis and inflammation: Role of caspase-3 and glycogen synthase kinase-3β enzymes. Life Sci.

[9] Lakka N, Pai B, Mani MS, Dsouza HS (2023). Potential diagnostic biomarkers for lead-induced hepatotoxicity and the role of synthetic chelators and bioactive compounds. Toxicol Res.

[10] Hoch CC, Petry J, Griesbaum L, Weiser T, Werner K, Ploch M (2023). 1, 8-cineole (eucalyptol): A versatile phytochemical with therapeutic applications across multiple diseases. Biomed Pharmacother.

[11] De Vincenzi M, Silano M, De Vincenzi A, Maialetti F, Scazzocchio B (2002). Constituents of aromatic plants: Eucalyptol. Fitoterapia.

[12] Galan DM, Ezeudu NE, Garcia J, Geronimo CA, Berry NM, Malcolm BJ (2020). Eucalyptol (1, 8-cineole): an underutilized ally in respiratory disorders?. J Essent Oil Res.

[13] Wang Y, Zhen D, Fu D, Fu Y, Zhang X, Gong G (2021). 1, 8-cineole attenuates cardiac hypertrophy in heart failure by inhibiting the miR-206-3p/SERP1 pathway. Phytomedicine.

[14] An F, Bai Y, Xuan X, Bian M, Zhang G, Wei C (2022). 1, 8-Cineole ameliorates advanced glycation end products-induced Alzheimer’s disease-like pathology in vitro and in vivo. Molecules.

[15] Mahdavifard S, Nakhjavani M (2022). 1, 8 cineole protects type 2 diabetic rats against diabetic nephropathy via inducing the activity of glyoxalase-I and lowering the level of transforming growth factor-1β. J Diabetes Metab Disord.

[16] Cai ZM, Peng JQ, Chen Y, Tao L, Zhang YY, Fu LY (2021). 1, 8-Cineole: A review of source, biological activities, and application. J Asian Nat Prod Res.

[17] Tang YL, Zhu L, Tao Y, Lu W, Cheng H (2023). Role of targeting TLR4 signaling axis in liver-related diseases. Pathol Res Pract.

[18] Meng C, Zeng W, Lv J, Wang Y, Gao M, Chang R (2021). 1, 8-cineole ameliorates ischaemic brain damage via TRPC6/CREB pathways in rats. J Pharm Pharmacol.

[19] Asiwe JN, Yovwin GD, Ekene NE, Ovuakporaye SI, Nnamudi AC, Nwangwa EK (2024). Ginkgo biloba modulates ET-I/NO signalling in Lead Acetate induced rat model of endothelial dysfunction: Involvement of oxido-inflammatory mediators. Int J Environ Health Res..

[20] ElBaset MA, Salem RS, Ayman F, Ayman N, Shaban N, Afifi SM (2022). Effect of empagliflozin on thioacetamide-induced liver injury in rats: role of AMPK/SIRT-1/HIF-1α pathway in halting liver fibrosis. Antioxidants.

[21] Lowry O, Rosebrough N, Farr AL, Randall R (1951). Protein measurement with the Folin phenol reagent. J Biol Chem.

[22] Jollow DJ, Mitchell JR, Zampaglione NA, Gillette JR (1974). Bromobenzene-induced liver necrosis Protective role of glutathione and evidence for 3, 4-bromobenzene oxide as the hepatotoxic metabolite. Pharmacology.

[23] Livak KJ, Schmittgen TD (2001). Analysis of relative gene expression data using real-time quantitative PCR and the 2−ΔΔCT method. Methods.

[24] Asle-Rousta M, Amini R, Aghazadeh S (2023). Carvone suppresses oxidative stress and inflammation in the liver of immobilised rats. Arch Physiol Biochem.

[25] Abdel Fattah ME, Sobhy HM, Reda A, Abdelrazek HM (2020). Hepatoprotective effect of Moringa oleifera leaves aquatic extract against lead acetate–induced liver injury in male Wistar rats. Environ Sci Pollut Res.

[26] Ciftci O, Ozdemir I, Tanyildizi S, Yildiz S, Oguzturk H (2011). Anti-oxidative effects of curcumin, β-myrcene and 1, 8-cineole against 2, 3, 7, 8-tetrachlorodibenzo-p-dioxin-induced oxidative stress in rats liver. Toxicol ind Health.

[27] Porres-Martínez M, González-Burgos E, Carretero ME, Gómez-Serranillos MP (2015). Major selected monoterpenes α-pinene and 1, 8-cineole found in Salvia lavandulifolia (Spanish sage) essential oil as regulators of cellular redox balance. Pharm Biol.

[28] Wang Y, Zhang X, Fu Y, Fu D, Zhen D, Xing A (2021). 1, 8-cineole protects against ISO-induced heart failure by inhibiting oxidative stress and ER stress in vitro and in vivo. Eur J Pharm.

[29] Cichoż-Lach H, Michalak A (2014). Oxidative stress as a crucial factor in liver diseases. World J Gastroenterol.

[30] Li S, Tan HY, Wang N, Zhang ZJ, Lao L, Wong CW (2015). The role of oxidative stress and anti-oxidants in liver diseases. Int J Mol Sci.

[31] Lugrin J, Rosenblatt-Velin N, Parapanov R, Liaudet L (2014). The role of oxidative stress during inflammatory processes. Biol Chem.

[32] Li W, Yang GL, Zhu Q, Zhong XH, Nie YC, Li XH (2019). TLR4 promotes liver inflammation by activating the JNK pathway. Eur Rev Med Pharmacol Sci.

[33] Kawasaki T, Kawai T (2014). Toll-like receptor signaling pathways. Front Immunol.

[34] Adachi O, Kawai T, Takeda K, Matsumoto M, Tsutsui H, Sakagami M (1998). Targeted disruption of the MyD88 gene results in loss of IL-1-and IL-18-mediated function. Immunity.

[35] Iacobazzi D, Convertini P, Todisco S, Santarsiero A, Iacobazzi V, Infantino V (2023). New Insights into NF-κB Signaling in Innate Immunity: Focus on Immunometabolic Crosstalks. Biology.

[36] Linghu K, Lin D, Yang H, Xu Y, Zhang Y, Tao L (2016). Ameliorating effects of 1, 8-cineole on LPS-induced human umbilical vein endothelial cell injury by suppressing NF-κB signaling in vitro. Eur J Pharmacol.

[37] Linghu KG, Wu GP, Fu LY, Yang H, Li HZ, Chen Y (2019). 1, 8-Cineole ameliorates LPS-induced vascular endothelium dysfunction in mice via PPAR-γ dependent regulation of NF-κB. Front Pharmacol.

[38] Lee HS, Park DE, Song WJ, Park HW, Kang HR, Cho SH (2016). Effect of 1 8-cineole in dermatophagoides pteronyssinus-stimulated bronchial epithelial cells and mouse model of asthma. Biol Pharm Bull.

[39] Lingappan K (2018). NF-κB in oxidative stress. Curr Opin Toxicol.

[40] Ramadori G, Moriconi F, Malik I, Dudas J (2008). Physiology and pathophysiology of liver inflammation, damage and repair. J Physiol pharmacol.

[41] Li Y, Xu YL, Lai YN, Liao SH, Liu N, Xu PP (2017). Intranasal co-administration of 1, 8-cineole with influenza vaccine provide cross-protection against influenza virus infection. Phytomedicine.

[42] McGill MR (2016). The past and present of serum aminotransferases and the future of liver injury biomarkers. EXCLI J.

[43] Santos FA, Silva RM, Tomé AR, Rao VS, Pompeu MM, Teixeira MJ (2001). cak protects against liver failure in an in-vivo murine model of endotoxemic shock. J Pharm Pharmacol.

[44] Kim NH, Hyun SH, Jin CH, Lee SK, Lee DW, Jeon TW (2004). Pretreatment with 1, 8-cineole potentiates thioacetamide-lnduced hepatotoxicity and immunosuppression. Arch Pharm Res.

[45] Akcakavak G, Kazak F, Yilmaz Deveci MZ (2023). Eucalyptol Protects against Cisplatin-Induced Liver Injury in Rats. Biol Bull Russ Acad Sci.

